# Down‐regulation of circDMNT3B is conducive to intestinal mucosal permeability dysfunction of rats with sepsis via sponging miR‐20b‐5p

**DOI:** 10.1111/jcmm.15324

**Published:** 2020-05-07

**Authors:** Jiao Liu, Yongan Liu, Lidi Zhang, Yizhu Chen, Hangxiang Du, Zhenliang Wen, Tao Wang, Dechang Chen

**Affiliations:** ^1^ Department of Critical Care Medicine Ruijin Hospital Shanghai Jiao Tong University School of Medicine Shanghai China; ^2^ Department of Critical Care Medicine Ruijin Hospital North Shanghai Jiao Tong University School of Medicine Shanghai China

**Keywords:** circDMNT3B, intestinal permeability, miR‐20b‐5p, sepsis

## Abstract

Sepsis is a life‐threatening syndrome with a high risk of mortality, which is caused by the dysregulated host response to infection. We examined significant roles of circDMNT3B and miR‐20b‐5p in the intestinal mucosal permeability dysfunction of rats with sepsis. SD rats were randomly divided into 6 groups (n = 10/group): sham group, sepsis group, si‐negative control group, circDNMT3B‐si1 group, circDNMT3B‐si2 group and circDNMT3B‐si1 + anti‐miR‐20b‐5p group. The level of malondialdehyde (MDA) content, superoxide dismutase (SOD) activity, interleukin (IL)‐6 and IL‐10 levels were measured through ELISA assay kits. Cell survival rate and cell apoptosis were evaluated by Cell‐Counting Kit‐8 Assay and flow cytometry, respectively. Luciferase reporter assays were used to investigate interactions between miR‐20b‐5p circDMNT3B in HEK‐293T cells. Silencing circDNMT3B can significantly increase the level of d‐lactic acid, FD‐40, MDA, diamine oxidase, IL‐10 and IL‐6, compared with sepsis group, while the SOD activity was lower. Silencing circDNMT3B leads to oxidative damage and influence inflammatory factors level in intestinal tissue. CircDNMT3B was identified as a target gene of miR‐20b‐5p. Silencing circDNMT3B decreased cell survival and induced apoptosis in Caco2 cells treated with LPS, which was reversed by anti‐miR‐20b‐5p. MiR‐20b‐5p inhibitor remarkably down‐regulated mentioned‐above levels, in addition to up‐regulate SOD activity, which may relieve the damage of intestinal mucosal permeability caused by silencing circDNMT3B in sepsis rats. Down‐regulation of circDMNT3B was conducive to the dysfunction of intestinal mucosal permeability via sponging miR‐20b‐5p in sepsis rats, which may provide the novel strategy for sepsis treatment in the future.

## 1 INTRODUCTION

Sepsis, a global health issue, is caused by a dysregulated host response to infection with a high risk of mortality rate.^1^ Sepsis is characterized by organ dysfunction, such as intestinal barrier dysfunction, haemodynamic instability and hypoxaemia.^2^ Prior studies suggested that anti‐inflammatory genes targeting relevant pathogenic factors and intestinal barrier dysfunction could reduce adverse inflammatory responses and bacterial translocation, thus increasing the survival rate for sepsis.^3^ Vitally, microRNAs (miRNAs) dysregulation was involved in clinical manifestations and inflammation, and they may be used as therapeutic targets for the treatment of sepsis.^4^

MiRNAs, 19‐25 nucleotides, are small non‐coding RNAs that modulate post‐transcriptional gene expression via specifically targeting mRNAs.^5^ Several miRNAs have been identified as potential biomarkers for the prognosis, diagnosis and treatment of sepsis.^6^, ^7^, ^8^ MiR‐20b‐5p originates from the conservative paralogous miR‐17‐2∼363 cluster ^9^ and have been reported its significant role in regulating cell differentiation, proliferation and apoptosis.^10^ Previous studies showed that miR‐20b‐5p was involved in the cell apoptosis, and the miR‐20b‐5p expression was significantly higher in the colorectal cancer group as compared to the normal group, which might be considered as a therapeutic biomarker.^11^ However, to our knowledge, the possible mechanisms of miR‐20b‐5p in sepsis progression are still unclear.

Circular RNAs (circRNAs) belong to a special class of non‐coding RNA, which is formed by a back splice mechanism through protein‐coding exons.^12^ Recently, growing evidence has suggested that circRNAs function as competing endogenous RNAs and miRNA sponges, and thus affecting the mRNA level in sepsis.^13^ CircRNAs and miRNAs are associated with anti‐inflammatory and pro‐inflammatory responses in sepsis.^14^, ^15^ In addition, increasing intestinal permeability may be related to sepsis progress in both Crohn's disease and ulcerative colitis patients.^16^, ^17^ CircDNMT3B was derived from the 8th and 9th exons of DNA methyltransferase 3 beta (DNMT3B).^18^ Zhu et al reported that decreased expression of circDNMT3B led to vascular dysfunction in diabetic retinas via modulating BAMBI and miR‐20b‐5p.^18^ However, the roles of circDNMT3B and miR‐20b‐5p in the development of intestinal permeability and sepsis are still not well discovered.

In this study, we have been suggested that circDNMT3B might be a target gene of miR‐20b‐5p and explored their significant role in the intestinal mucosal permeability dysfunction of rats with sepsis, aiming to provide important insights into the clinical diagnosis and treatment of sepsis.

## 2 METHODS

### 2.1 The establishment of cecum ligation and perforation rats model

Sprague‐Dawley male rats weighing 240‐340 g were obtained from Shanghai SLAC Laboratory Animal Co., Ltd. The rats were acclimated for 7 days before the experiment. The cecum ligation and perforation (CLP) sepsis model was conducted as follows. Firstly, a median abdominal incision (2 cm) was made to open the abdominal cavity. Next, the cecum and proximal colon were ligated 0.5 cm under the ileocecal valve with 1.0 silk suture and were punctured on the mesenteric border with 8‐gauge needles. We left 1.0 silk suture to avoid pinholes closing. Sham rats only received dissociation, without perforation and ligation. After the operation, all rats were injected with 50 mL/kg Ringer's solution subcutaneously to replenish fluid. The CLP model was considered successful if positive blood intestinal microflora results and clinical sepsis signs appeared six hours after the CLP operation.^19^ All animal experiments were approved by the Animals Ethics Committee of Shanghai Jiaotong University, School of Medicine, Ruijin Hospital North.

### 2.2 Animal grouping and treatment

The rats were randomly divided into 6 groups (n = 10/group): sham group, sepsis group, si‐negative control (NC) group, circDNMT3B‐si1 group, circDNMT3B‐si2 group and circDNMT3B‐si1 + anti‐miR‐20b‐5p group. The treatment in each group was listed in Table 1. The surgery was carried out based on the instructions.

**TABLE 1:** The animal grouping in this study

Group	Model	Treatment (intravenous injection 24 h before the operation)
sham	sham	1 mL normal saline
sepsis CLP model rats	CLP model rats	1 mL normal saline
si‐NC	CLP model rats	10 μg NC sequence
circDNMT3B‐si1	CLP model rats	10 μg circDNMT3B‐si
circDNMT3B‐si2	CLP model rats	10 μg circDNMT3B‐si
circDNMT3B‐si1 + anti‐miR‐20b‐5p	CLP model rats	10 μg circDNMT3B‐si and anti‐miR‐20b‐5p

Abbreviations: CLP, cecum ligation and perforation; NC, negative control.

### 2.3 Specimen collection

Five rats from each group were anaesthetized via intraperitoneal injection of 1% pentobarbital (0.2 mL/100 g), 24 hours after the operation. Next, blood was obtained from the abdominal aorta, and serum was stored at −80°C after separation. Under sterile conditions, the intestinal tissues were incised and preserved at −80°C until analysis.

### 2.4 Content of malondialdehyde and the activity of superoxide dismutase

HCI (1 mol/L) solutions were added to intestinal tissues collected from the 24 hours after CLP or sham group to prepare tissue homogenates. To obtain the supernatant, centrifugation followed at 1812 × *g* for 10 minutes. The assay kits for the malondialdehyde (MDA) content and the superoxide dismutase (SOD) activity were ordered from the Nanjing Jiancheng Bioengineering Institute (Nanjing, China). The detection was under the instructions of manufactures.

### 2.5 Inflammatory factors detection

A homogenizer was used to treat the intestinal tissues collected from the 24 hours after CLP or sham group. After centrifugation for 15 minutes at 20 128 × g, the supernatant was collected. The interleukin (IL)‐6 and IL‐10 levels were measured through ELISA assay kits purchased from Shanghai Tong Wei Biological Technology Co., Ltd (Shanghai, China). All steps were conducted strictly according to the instructions and previously described methods.^20^

### 2.6 Intestinal mucosal permeability assay

AMPLITE™ colorimetric d‐lactic acid test kit (Biolite Biotech, China) measured the d‐lactic acid levels in serum. Diamine oxidase (DAO) assay kit purchased from Nanjing Jiancheng Bioengineering Institute (Nanjing, China) detected the serum DAO levels in rats. All specific operations were conducted followed the kit instructions.

After the operation for 18 hours, 750 mg/kg FD‐40 was gavage‐administered to rats in each group. Venous blood samples were taken after 6 hours of gavage administration, and then the serum was separated. The absorbance was detected at the excitation wavelength of 490 nm and emission of 520 nm to evaluate the FD‐40 serum levels. Parallel experiments were repeated three times, and the mean value was reported.

### 2.7 Cell culture

We obtained HEK‐293T cells and Caco2 cells from the Chinese Academy of Sciences (Shanghai, China). Cells were cultured in DMEM high sugar medium (Gibco, USA), which contained 10% FBS, 2.5 mg/mL plasmocin and 1% non‐essential amino acids at 37℃ with 5% carbon dioxide. Caco2 cells were treated with LPS (Solarbio, China).

### 2.8 Cell‐Counting Kit‐8 Assay

The cell survival rate was assessed via CCK‐8 (Dojindo Laboratories, Japan) based on the instructions. Briefly, Caco2 cells were seeded into a 96‐well plate at a density of 1 × 10^4^ per well. Next, 10 μL of CCK‐8 reagent (2.5 g/L) was added to each well, and cells were fostered for two hours. Finally, the absorbance was measured at a wavelength of 450 nm utilizing a microplate reader (Molecular Devices, USA).

### 2.9 Cell proliferation assay

The EdU kit (RiboBio, China) was applied to detect cell proliferation. In short, 50 μmol/L EdU medium was added to each well and Caco2 cells were fostered at 37°C for two hours. Then EdU was employed to fix and stain cells. Images were taken through a fluorescence inverted microscope.

### 2.10 Cell apoptosis

The Annexin V‐FITC/PI apoptosis assay kit (BD Biosciences, USA) was utilized to evaluate cell apoptosis. Firstly, Caco2 cells were centrifuged at 168*g* for 5 minutes and washed twice with PBS. Then 300 μL 1xAnnexin‐binding buffer was added to each centrifuge tube to resuspend cells. Next, we added Annexin V (5 μL) and PI (1 μL, 100 μg/mL) to the cell suspension. Subsequently, cells were fostered for 15 minutes in the dark. Eventually, the apoptosis rate was assessed via BD FACS software.

### 2.11 Haematoxylin and eosin staining

For histopathological examination, 4% formaldehyde was utilized to fix the middle parts of rats’ intestinal tissues for six hours. Next, tissues were embedded in paraffin, cut into 5 μm‐thick sections, and stained with haematoxylin and eosin (HE) as previously reported.^21^

### 2.12 Double luciferase assay

Lipofectamine 2000 was employed to co‐transfect HEK‐293T cells with the luciferase report vector, containing the 3′UTR of WT‐circDNMT3B or MUT‐circDNMT3B and miR‐20b‐5p mimic or mimic‐NC. After HEK‐293T cells transfection of 24 hours, the luciferase activity was detected based on the manufacturer's instructions with the Luciferase Assay Reporter System (Promega, Madison, WI, USA).

### 2.13 Western blot assay

Western blot was carried out based on the manufacturer's protocol. Bcl‐2 (1:1000), BAX (1:1000) antibodies and GAPDH (1:1000) were purchased from Abcam (Cambridge, UK).

### 2.14 Statistical analysis

All experimental data, represented as in mean ± standard deviation, were compared utilizing SPSS 22.0. The two‐tailed *t* test was used to compare differences between two groups, and one‐way ANOVA followed by Bonferroni's multiple comparisons test for more than three groups. *P* < 0.05 was considered as statistically significant.

## 3 RESULTS

### 3.1 The role of circDNMT3B‐si in the permeability of intestinal mucosal

**FIGURE 1:**
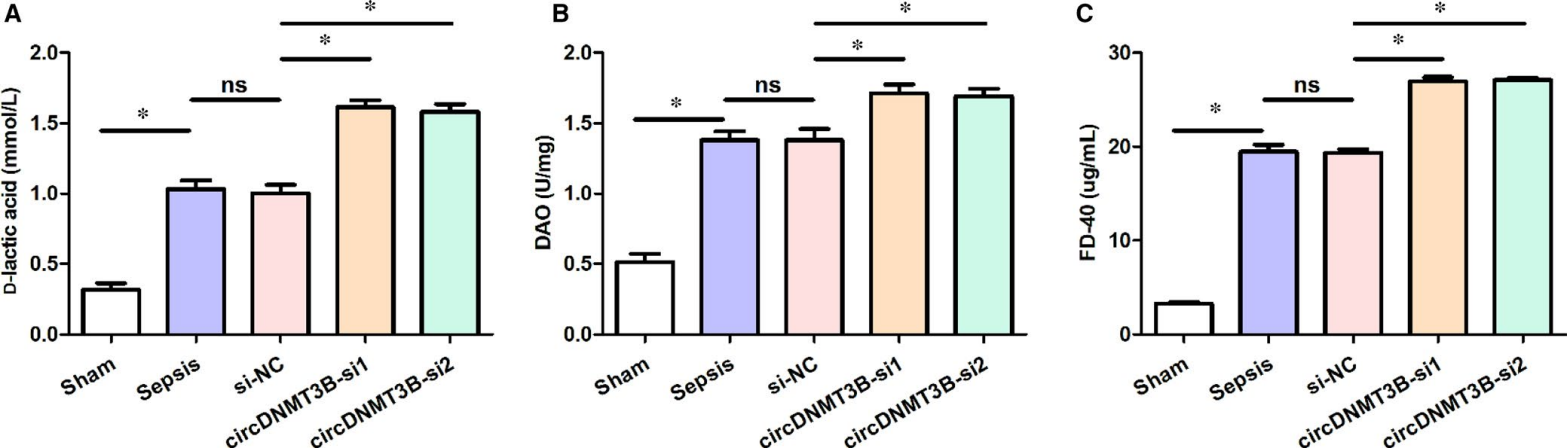
The permeability of intestinal mucosal was affected by silencing circDNMT3B. A, Serum levels of d‐lactic acid elevated after silencing circDNMT3B; B, serum levels of DAO up‐regulated by silencing circDNMT3B; C, serum levels of FD‐40 increased after silencing circDNMT3B; *, *P* < 0.05, ns: not significant; NC, negative control; DAO, diamine oxidase

To explore the effect of circDNMT3B in the permeability of intestinal mucosal, rats received CPL operation and administration to establish the sepsis model during the study period. Then, we collected serum samples 24 hours after the injection to measure the levels of d‐lactic acid (mmol/L), FD‐40 (µg/mL) and DAO (U/mg) in serum. As can be seen from Figure 1, the serum d‐lactic acid (Figure 1A), DAO (Figure 1B) and FD‐40 (Figure 1C) levels were significantly increased in the other four groups, compared with the sham group. Moreover, rats in the circDNMT3B‐si group had remarkably higher serum levels of d‐lactic acid, DAO and FD‐40 than those of the sepsis group.

### 3.2 Silencing circDNMT3B can lead to oxidative damage and influence inflammatory factors level in intestinal tissue

**FIGURE 2:**
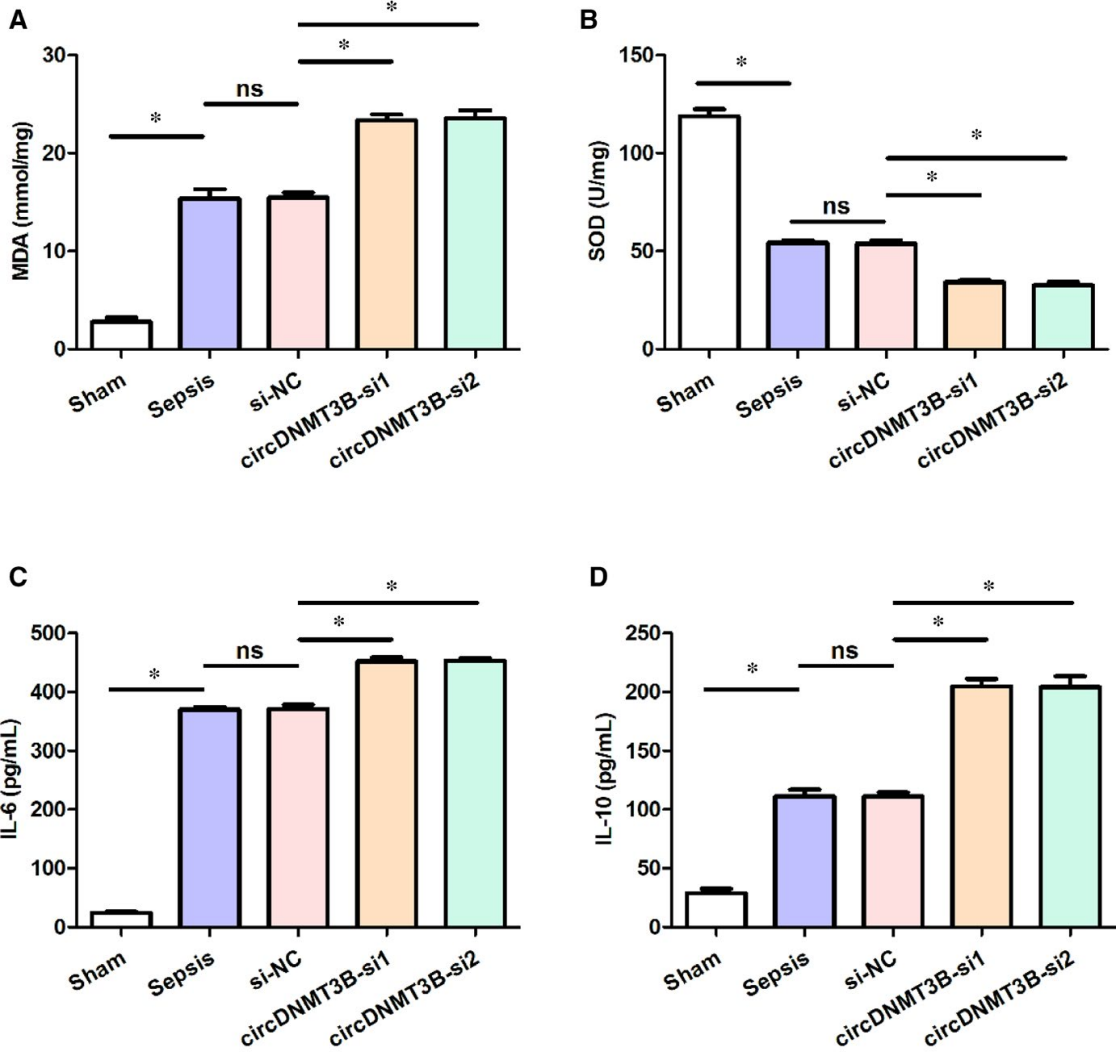
Silencing circDNMT3B caused oxidative damage and influenced inflammatory factors level in intestinal tissue. A, The content of MDA elevated after silencing circDNMT3B; B, SOD activity decreased by silencing circDNMT3B; C, up‐regulated levels of IL‐6 after silencing circDNMT3B; D,, increased levels of IL‐10 by silencing circDNMT3B; *, *P* < 0.05, IL, interleukin; MDA, malondiadehyde; NC, negative control; ns, not significant; SOD, superoxide dismutase

To evaluate the effect of silencing circDNMT3B on oxidative damage, the content of MDA (mmol/mg) and the activity of SOD (U/mg) were determined in rats’ intestinal tissues. As shown in Figure 2A,B, compared with the sham group, there was a significant increase in the MDA level, but an evident decrease in the SOD activity in the other four groups. Besides, the circDNMT3B‐si group reported significantly more MDA content and less SOD activity than the sepsis group.

In addition, we measured IL‐6 (pg/mL) and IL‐10 (pg/mL) levels to investigate the inflammation caused by silencing circDNMT3B in the intestinal tissue of rats. As presented in Figure 2C,D, the levels of IL‐6 and IL‐10 in the other four groups were significantly higher compared with those of the sham group. Furthermore, the remarkably increased contents of IL‐6 and IL‐10 were observed in the circDNMT3B‐si group, compared with the sepsis group. These results indicated that silencing circDNMT3B could induce oxidative damage and increase inflammatory factors level in intestinal tissue.

### 3.3 Silencing circDNMT3B decreased cell survival and induced apoptosis in Caco2 cells treated with LPS

**FIGURE 3:**
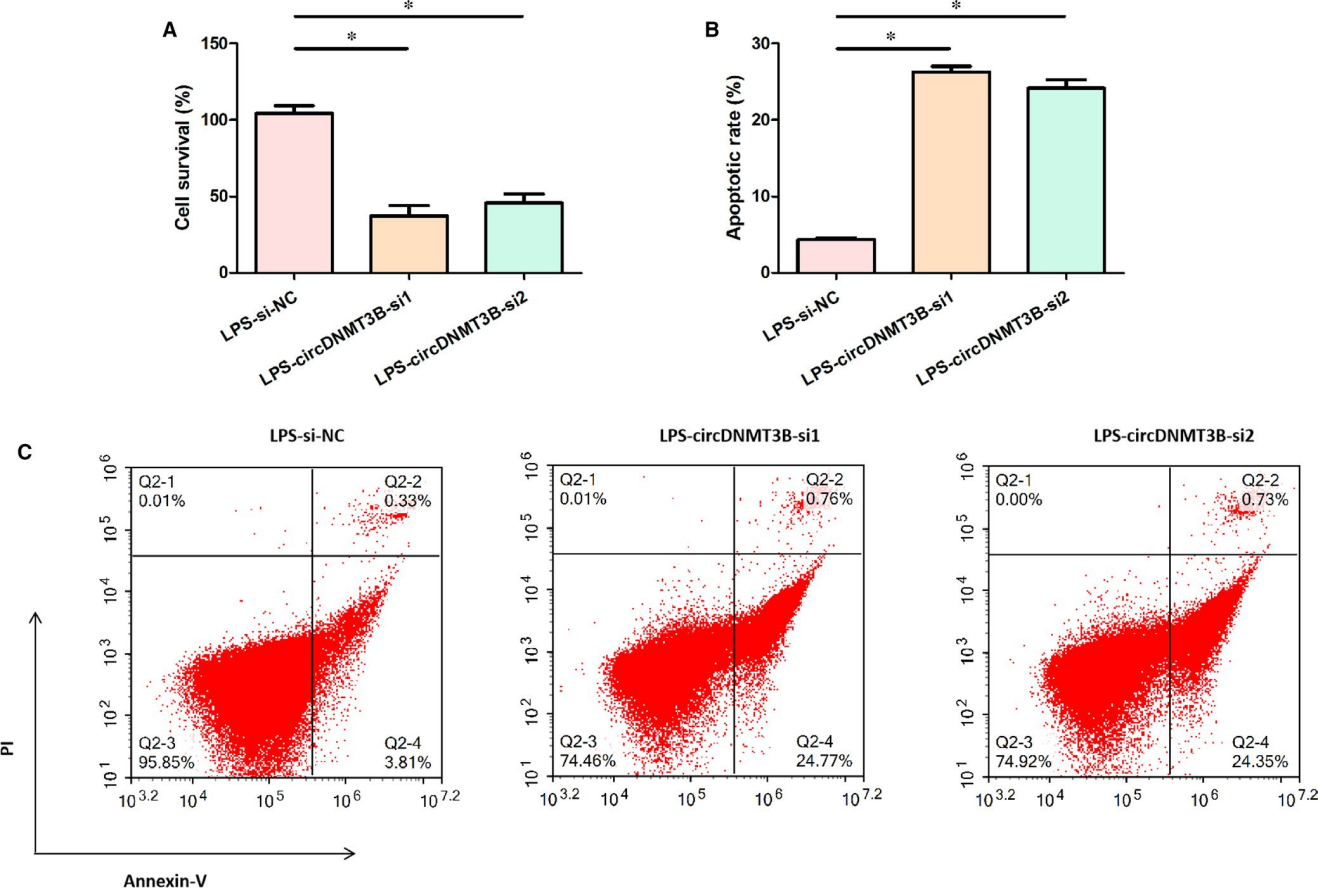
Silencing circDNMT3B decreased cell survival and induced apoptosis in Caco2 cells treated with LPS. A, Cell survival of LPS‐treated Caco2 cells was determined by CCK‐8 assay; B and C, cell apoptosis of LPS‐treated Caco2 cells was measured by flow cytometry assay; *, *P* < 0.05, NC, negative control

As displayed in Figure 3A, cell survival was significantly reduced after silencing circDNMT3B in LPS‐treated Caco2 cells. The results of flow cytometry assay in Figure 3B,C indicated that the apoptotic rate of LPS‐treated Caco2 cells was remarkably elevated via silencing circDNMT3B. These results suggested that silencing circDNMT3B reduced cell survival and induced apoptosis in LPS‐treated Caco2 cells.

### 3.4 CircDNMT3B was identified as a target gene of miR‐20b‐5p

**FIGURE 4:**
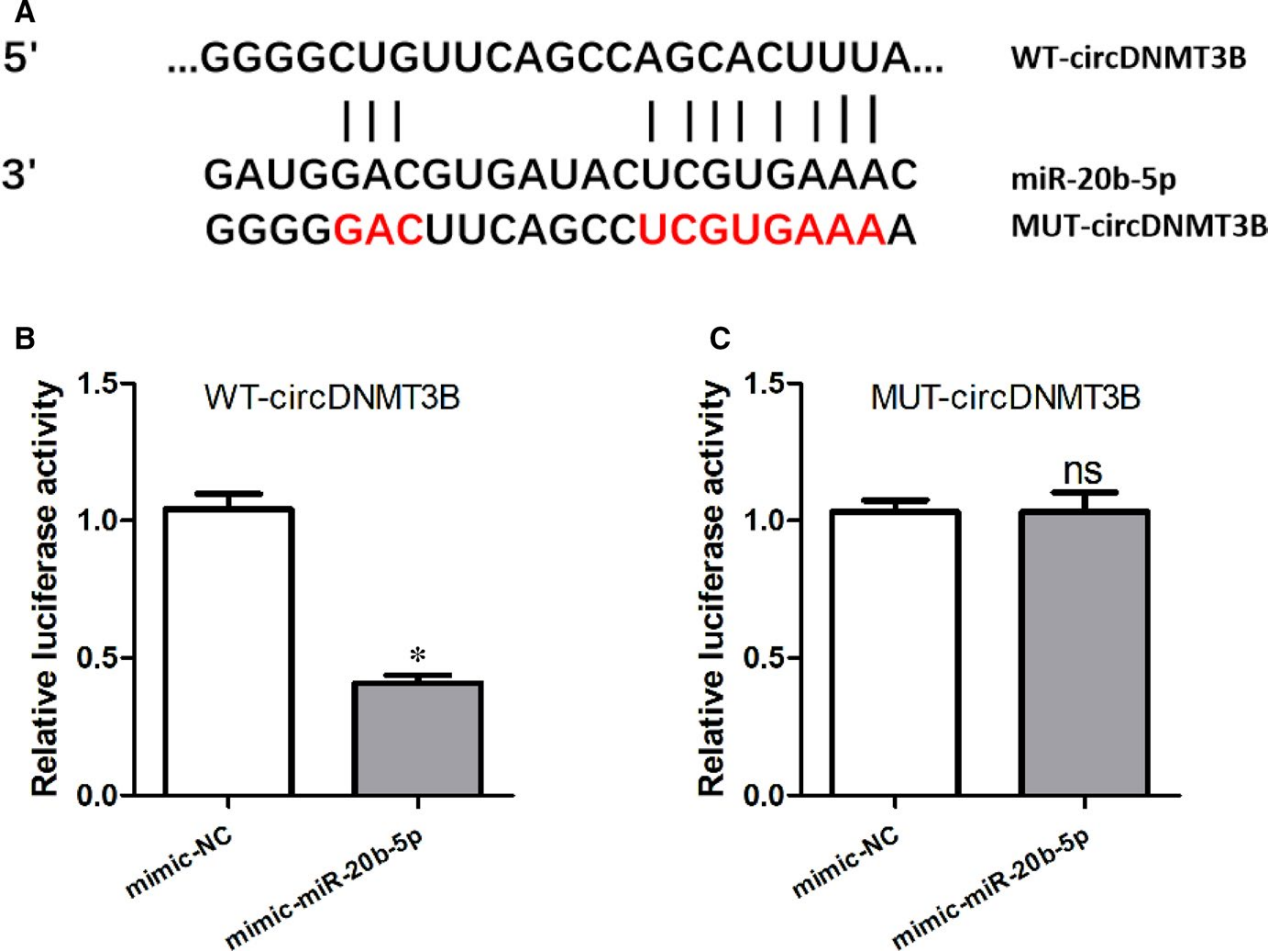
CircDNMT3B was identified as a target gene of miR‐20b‐5p. A, CircDNMT3B was identified as a target gene of miR‐20b‐5p by the microRNA.org website; B and C, relative luciferase activities in HEK‐293T cells after transfection with the miR‐20b‐5p mimic, mimic‐NC containing either circDNMT3B wild type (WT) (B) or mutant type (MUT) (C); *, *P* < 0.05, NC, negative control

CircDNMT3B was identified as the miR‐20b‐5p target gene through the microRNA.org website, as shown in Figure 4A. The relative luciferase activity was remarkably lower in the mimic‐miR‐20b‐5p + WT‐circDNMT3B group than that in the mimic‐NC + WT‐circDNMT3B group, as displayed in Figure 4B. However, Figure 4C indicated that no significant differences in luciferase activity were found between the mimic‐miR‐20b‐5p + MUT‐circDNMT3B group and mimic‐NC + MUT‐circDNMT3B group. These data revealed that miR‐20b‐5p might target circDNMT3B.

### 3.5 The effect of miR‐20b‐5p on the permeability of intestinal mucosal via modulating circDNMT3B

**FIGURE 5:**
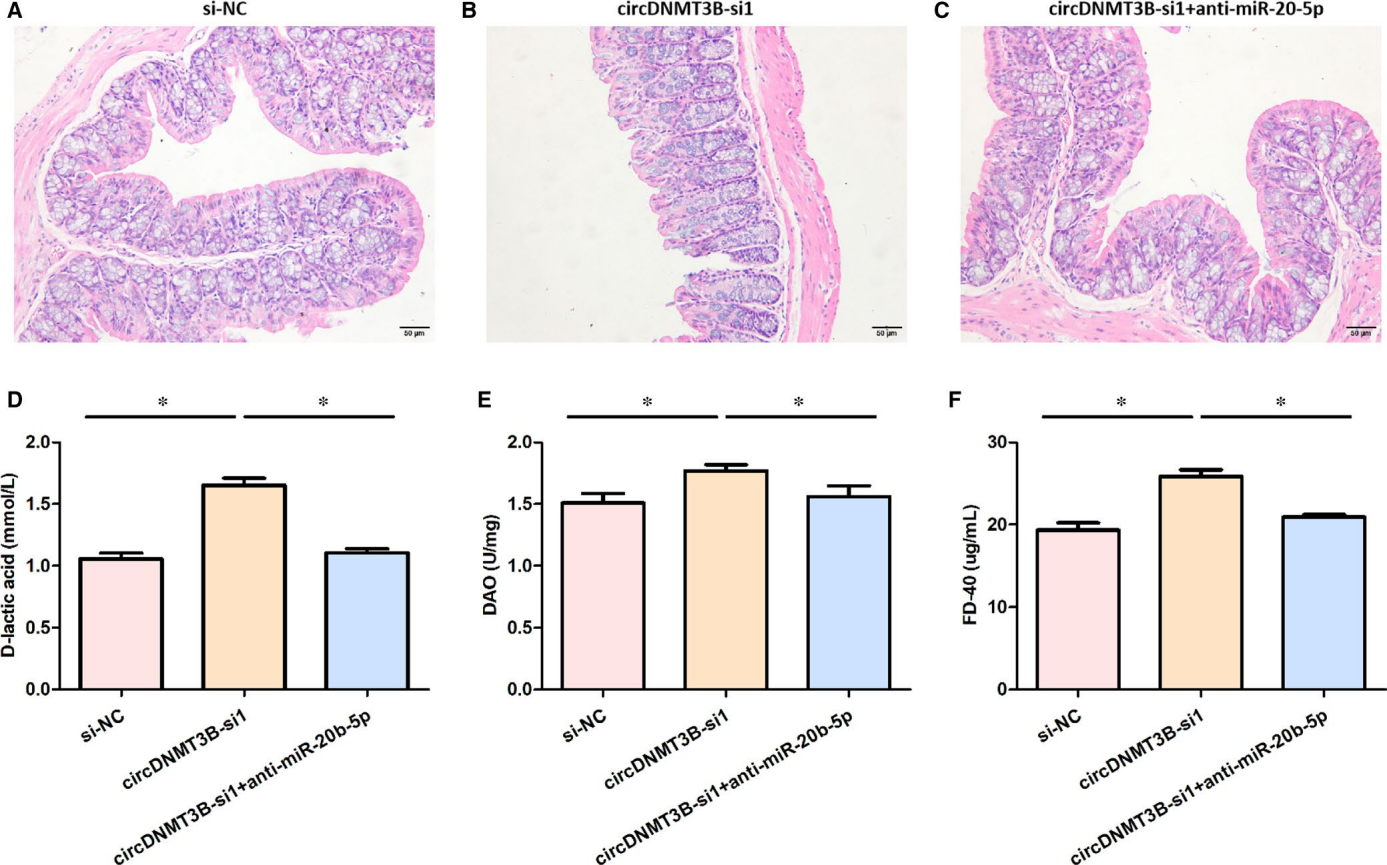
MiR‐20b‐5p might influence intestinal mucosa permeability through regulating circDNMT3B. A‐C, Histopathological changes of the intestine tissues of the rats in each group; D, serum levels of d‐lactic acid in three groups; E, serum levels of DAO in three groups; F, serum levels of FD‐40 in three groups; *, *P* < 0.05; DAO, diamine oxidase; NC, negative control

CircDNMT3B was interfered with anti‐miR‐20b‐5p to explore the effect of miR‐20b‐5p on the permeability of intestinal mucosal via modulating circDNMT3B. As demonstrated in Figure 5A,B, the HE staining results revealed that the intestinal wall and intestinal mucosa of rats in the circDNMT3B‐si1 group were more atrophy, and showed more intestinal mucosa necrosis and shedding, compared with the si‐NC group. In the circDNMT3B‐si1 + anti‐miR‐20b‐5p group, some intestinal mucosa necrosis and shedding occurred, but the intestinal mucosa injury was relatively mild in Figure 5C. As presented in Figure 5D‐F, the serum levels of d‐lactic acid, FD‐40 and DAO in the circDNMT3B‐si1 group were significantly higher than that of the si‐NC group. However, the serum levels of d‐lactic acid, FD‐40 and DAO were remarkably decreased in the circDNMT3B‐si1 + anti‐miR‐20b‐5p group, compared with the circDNMT3B‐si1 group. These findings indicated that the permeability of intestinal mucosal might be influenced by miR‐20b‐5p via regulating circDNMT3B.

### 3.6 MiR‐20b‐5p influenced oxidative damage and the levels of inflammatory factors in intestinal tissue via circDNMT3B

**FIGURE 6:**
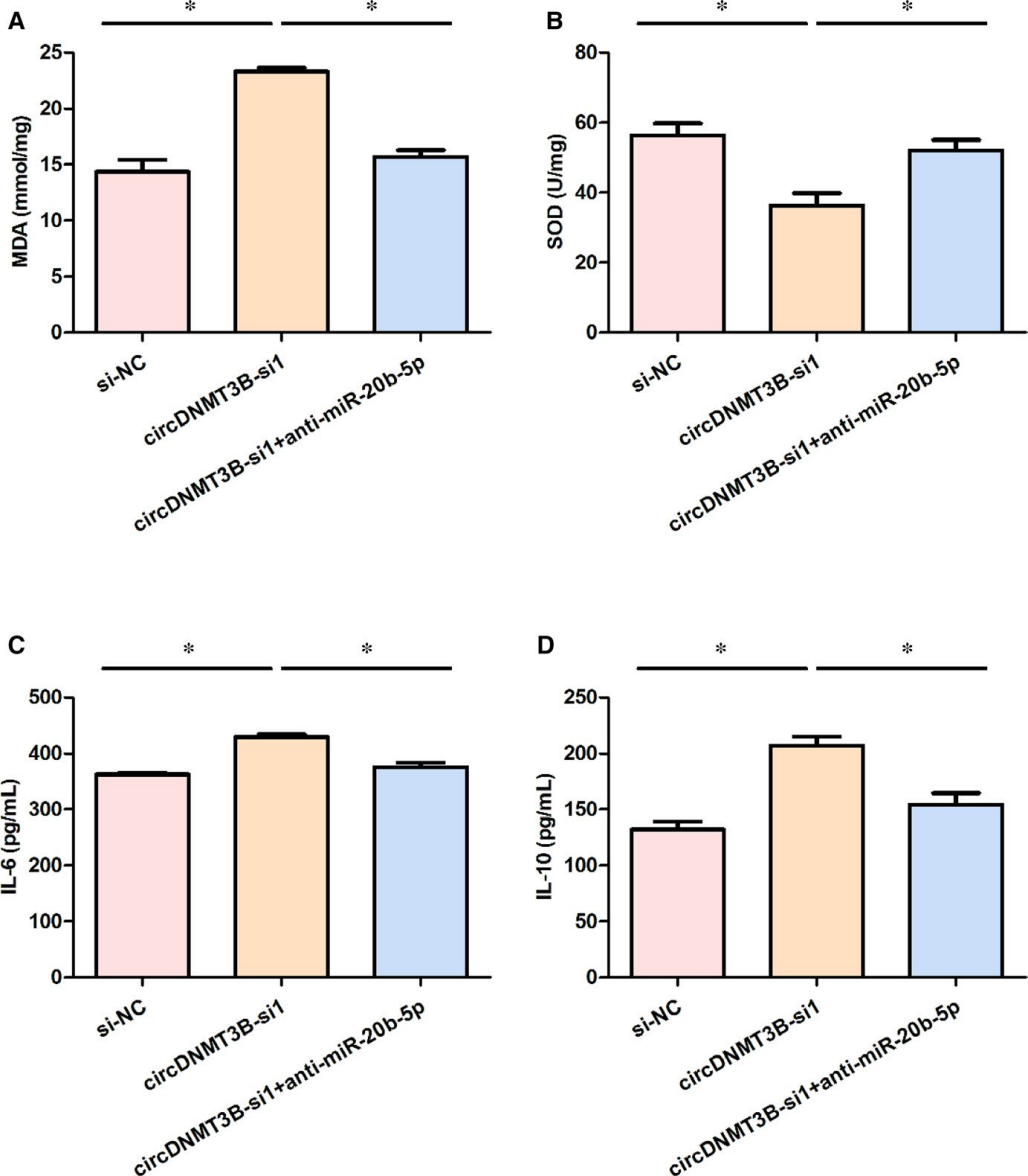
MiR‐20b‐5p influenced oxidative damage and the levels of inflammatory factors in intestinal tissue via circDNMT3B. A, The content of MDA in three groups; B, SOD activity in three groups; C, levels of IL‐6 in three groups; D, levels of IL‐10 in three groups; *, *P* < 0.05; IL, interleukin; MDA, malondfundsinadehyde; NC, negative control; SOD, superoxide dismutase

From the data in Figure 6A,B, it can be seen that the circDNMT3B‐si1 group had a significantly increased MDA level and decreased SOD activity compared with the si‐NC group, while the circDNMT3B‐si1 + anti‐miR‐20b‐5p group had a significantly lower MDA level and higher SOD activity than the circDNMT3B‐si1 group.

Furthermore, as presented in Figure 6C,D, the IL‐6 and IL‐10 levels were significantly higher in the circDNMT3B‐si1 group than in the si‐NC group. However, the IL‐6 and IL‐10 levels in the circDNMT3B‐si1 + anti‐miR‐20b‐5p group decreased evidently compared with those in the circDNMT3B‐si1 group. These results suggested that miR‐20b‐5p could lead to oxidative damage in the small intestine of rats and influence inflammation levels via circDNMT3B.

### 3.7 MiR‐20b‐5p inhibitor enhanced proliferation and suppressed apoptosis of Caco2 cells treated with LPS and silencing circDNMT3B

**FIGURE 7:**
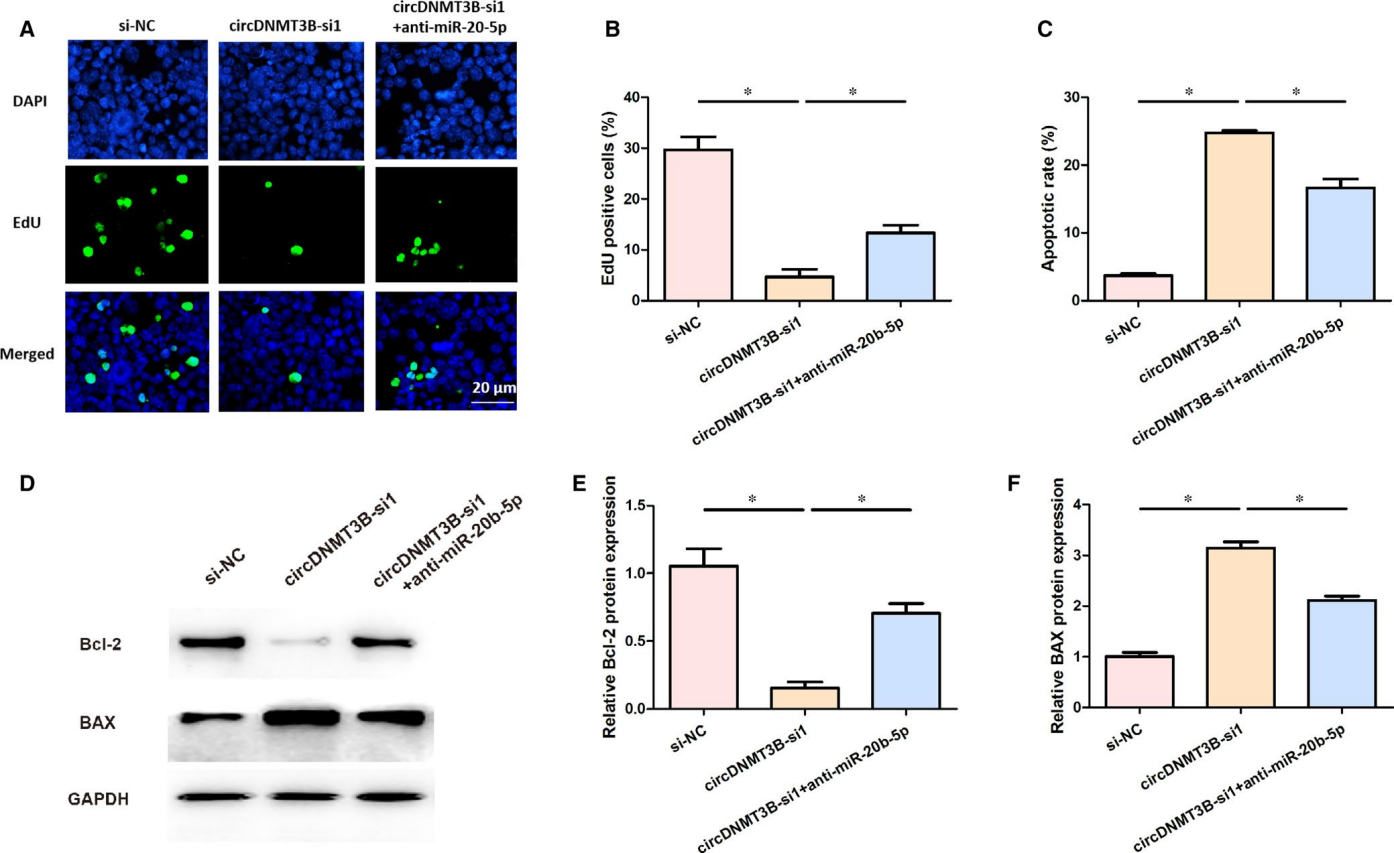
MiR‐20b‐5p inhibitor enhanced proliferation and suppressed apoptosis of Caco2 cells treated with LPS and silencing circDNMT3B. A and B, Cell proliferation of Caco2 cells was determined by EdU assay; C, cell apoptosis of Caco2 cells was measured by flow cytometry assay; D, Bcl‐2 and BAX protein expression in each group; E and F, relative Bcl‐2 and BAX protein expression in each group. *, *P* < 0.05, NC, negative control

As can be seen from Figure 7A‐C, silencing circDNMT3B was significantly reduced cell proliferation and induced apoptosis in LPS‐treated Caco2 cells, which was reversed by anti‐miR‐20b‐5p. As shown in Figure 7D‐F, silencing circDNMT3B could remarkably repressed Bcl‐2 protein expression and increased BAX protein expression, which was reversed via anti‐miR‐20b‐5p in LPS‐treated Caco2 cells. These data suggested that miR‐20b‐5p inhibitor enhanced proliferation and suppressed apoptosis of Caco2 cells treated with LPS and silencing circDNMT3B.

## 4 DISCUSSION

Sepsis is a complex and life‐threatening syndrome with a high risk of mortality, which is caused by the dysregulated host response to infection.^22^ It has been reported that increased intestinal permeability may lead to the development of sepsis in both Crohn's disease and ulcerative colitis patients.^16^, ^17^ Previous studies showed that the dysfunction of miRNAs was associated with the development of intestinal permeability and sepsis.^8^, ^23^ In the current study, we found that the down‐regulation of circDMNT3B was conducive to intestinal mucosal permeability dysfunction of rats with sepsis via sponging miR‐20b‐5p.

In the present study, our findings indicated that rats with sepsis showed higher levels of d‐lactic acid, FD‐40, MDA, DAO, IL‐10 and IL‐6, compared with the sham group, but the SOD activity was decreased in sepsis rats. Our results were supported by the Zhang et al study, which showed that the sepsis group reported a significantly higher serum d‐lactic acid level than that in the healthy control group.^24^ Likewise, the activity of SOD was increased, while the content of MDA was decreased in pancreatitis rats with intestinal barrier dysfunction.^25^ Intestinal barrier dysfunction caused translocation of intestinal bacteria and induced numerous endogenous inflammatory mediators, which reduced the intestinal mucosa's own antioxidant capacity. Therefore, the level of MDA increased and the activity of SOD decreased in the small intestine and serum of rats with pancreatitis.^25^ Also, prior studies observed that serum IL‐6 and IL‐10 levels were remarkably up‐regulated in patients with sepsis.^26^, ^27^ Sepsis is mostly initiated by the inflammatory cascade.^1^ The early manifestation is the increase of pro‐inflammatory cytokines represented by IL‐6, producing different degrees of inflammatory reactions, which are accompanied by the body's anti‐inflammatory response.^27^ IL‐10 plays a vital role in down‐regulating pro‐inflammatory cytokines and reducing uncontrolled inflammatory responses, which may help reduce tissue damage and improve the outcomes of sepsis patients.^26^

In addition, our data revealed that silencing circDNMT3B could contribute to the significantly increased level of d‐lactic acid, FD‐40, MDA, DAO, IL‐10 and IL‐6, in comparison to the sepsis group, while the activity of SOD was lower. Therefore, silencing circDNMT3B may contribute to the dysfunction of intestinal mucosal permeability in rats with sepsis. However, miR‐20b‐5p inhibitor remarkably down‐regulated mentioned‐above levels, in addition to up‐regulate SOD activity, which may relieve the damage of intestinal mucosal permeability caused by silencing circDNMT3B in sepsis rats. Accumulating evidence demonstrates that circRNAs serve significant roles in sepsis via targeting miRNA.^13^, ^28^ Previously, a recent study observed that suppression of miR‐31 could protect against intestinal barrier dysfunction of sepsis rats by inhibition of the NF‐κB/HIF‐1α pathway via sponging HMOX1.^20^ This study indicated that miR‐20b‐5p was the circDNMT3B direct target, and further this association was verified by the luciferase report assay. This finding was supported by the study suggested that miR‐506 and miR‐124 inhibited the progression of colorectal cancer via regulating DNMT1B and DNMT3.^29^ Moreover, another study showed that the down‐regulation of MALAT1 could suppress tumorigenesis through modulating miR‐20b‐5p/Oct4 network in colorectal cancer.^30^ These results from our study revealed that circDNMT3B may act as a miR‐20b‐5p sponge, negatively modulating the level of miR‐20b‐5p in rats with sepsis.

In conclusion, our findings indicated that the down‐regulation of circDMNT3B was conducive to the dysfunction of intestinal mucosal permeability via sponging miR‐20b‐5p in rats with sepsis, which may offer new insights into sepsis and provide the novel strategy for sepsis treatment in the future.

## CONFLICT OF INTEREST

The authors confirm that there are no conflicts of interest.

## AUTHOR CONTRIBUTION

Jiao Liu and Yongan Liu contributed to experimental design and writing the manuscript. Lidi Zhang, Yizhu Chen and Hangxiang Du conducted the experiments. Zhenliang Wen and Tao Wang performed the statistical analysis. Dechang Chen had overall responsibility for the present study. All authors approved the final manuscript version.

## Data Availability

Data available on request from the authors.
